# Perceived impact of field trips on students’ sense of community, skills and knowledge in Biosciences and Chemistry undergraduate degrees

**DOI:** 10.1042/ETLS20253024

**Published:** 2026-04-22

**Authors:** Melissa M. Lacey, Neil Bricklebank, David P. Smith, Rachel Schwartz-Narbonne

**Affiliations:** 1 School of Biosciences and Chemistry, Sheffield Hallam University, Sheffield, U.K.

**Keywords:** 14-3-3 proteins, AAA proteins, ABC transport proteins, abdominal aortic aneurysm, accessory pigments

## Abstract

Field trips are widely recognised for their pedagogical value in natural sciences, yet their role in Biosciences and Chemistry undergraduate education remains underexplored. This case study investigates the perceived impact of field trips on students’ subject knowledge, employability skills and sense of community within the School of Biosciences and Chemistry at Sheffield Hallam University. Using questionnaire data from students and academic staff across five undergraduate programmes, the study reveals that field trips are highly valued for linking theory to practice, strengthening practical and employability skills, and fostering peer relationships. Biology students expressed the strongest desire for field trips, while Biochemistry students showed the least interest. Academic staff, and the majority of undergraduates, supported field trip inclusion across all courses. Thematic analysis of questionnaire open-text responses identified key benefits, including deepened subject knowledge, career insights and increased course belonging. However, barriers to participation – primarily financial constraints, health concerns and work commitments – were reported by both students and staff. Recommendations to improve accessibility included offering free or low-cost trips, scheduling within teaching hours and early communication. The findings underscore the importance of inclusive, well-structured field trip opportunities to enhance student engagement and learning outcomes. This research informs the development of a cost-effective and accessible field trip programme across Biosciences and Chemistry courses, aligning with institutional goals and student needs.

## Introduction

Instructional trips outside the classroom, such as via field trips to conduct fieldwork, are a common component of Geography, Biology, Geology and Environmental Sciences University degrees [1–6]. These trips are known to benefit students on multiple axes, including subject-specific content and skills gained through active learning, knowledge co-creation and authentic learning experiences, as well as benefiting students’ interpersonal skills and social connections [7]. In addition to their pedagogical benefits, institutions are motivated to include field trips in these courses by accreditation requirements and subject-specific expectations. For example, the British Ecological Society recommends practical fieldwork skills for graduates of ecology degree programmes [8].

There is less pedagogical literature on field trips in university Bioscience and Chemistry courses besides Biology. This likely stems from the lack of fieldwork requirements for accreditation of these courses by the IBMS, RSB and RSC, alongside institutional barriers to running field trips (e.g. increasing class sizes, financial costs, legal liability) [7,9]. However, field trips are not limited to fieldwork activities conducted within natural environments. Krepel and Duvall [9] define field trips as school-arranged trips with an educational intent where students interact with settings, displays and exhibits to gain experiential connections [9]. Tal and Morag [10] describe field trips as non-classroom-based, educational and interactive [10]. Outdoor learning, such as fieldwork, comprises a subset of these out-of-school learning opportunities, which may additionally include visits to museums, community organisations, libraries, science centres, etc. [7,10,11]. These forms of field trips will be more relevant to other Bioscience and Chemistry Courses, which, unlike Biology, do not typically involve field work as part of their practical skills.

Field trips are suggested to have a range of educational benefits, both directly related to course content and more broadly. As a pedagogical tool, they promote the co-creation of knowledge, active learning, experiential learning, real-world place-based learning and rapid feedback from instructors, which combine to produce higher-order forms of learning from Bloom’s Taxonomy. They also promote peer-to-peer interactions and feedback, promoting social connections [1,2,7,11–13]. Where field trips are used outside environmental-based courses, literature suggests similar benefits accrue to students. For example, Biomedical Science students at the University of Minnesota visited a medical library with historical artefacts, situating them in course contexts [14]. An interdisciplinary module at De Montfort University took students, including Biomedical Science students, on an international trip, benefiting their transferable skills, including teamwork and communication [15]. Natural sciences were taught on field trips to Chemistry, Biology and Biochemistry students at the University of Timişoara, deepening and broadening their content knowledge [16]. Field trips to research and diagnostic centres increased biomedical science students’ understanding of their subject at a Malaysian university; these locations were selected based on relevance to taught modules [13].

Full participation in course trips is not equally available to all students. Those with physical disabilities have reported their access needs not being met in field work environments, and those with mental health issues and neurodiversity can find the high social interactions often associated with field trips overwhelming [1,17–19]. Innovative practice to increase the accessibility of field trips includes the provision of virtual field trips and self-guided field trips [1,20,21].

## Educational context

The School of Biosciences and Chemistry is composed of five courses: Chemistry (~25 students per cohort), Biochemistry (~20 students per cohort), Biology (~20 students per cohort), Biomedicine and Health Science (~20 students per cohort) and Biomedical Science (~100 students per cohort).

Within this work, a field trip is defined as a department-led activity outside of the university campus/premises. The academic year this study was undertaken (Sept 2023 to May 2024), students on three of these courses attended field trips which were designed for their discipline: Biology (in first and second year); Biomedical Sciences (in second year) and Chemistry (in second year), while Biochemistry and Biomedicine and Health Science courses did not attend field trips. All students reflected on their second-year trips within their end-of-year E-Portfolio assessments and received feedback on their reflections. Biology students also wrote a group work assessment on their findings from their first-year field trip. Additional field trips were under development for all courses to further provide students with skills and knowledge and to promote a sense of community. The study was motivated by the need to assess students’ and staff’s perceived benefits of field trips to support this development.

## Methods

### Questionnaire design

The questionnaire was designed to assess students’ and staff’s perceived benefits of field trips to support this development. Questions were chosen to allow bivariate analysis of responses by course. Questions were mirrored in the academic staff and student questionnaires to allow comparison between the two groups.

### Participants and data collection

All BSc undergraduate students (~550) and academic staff (~45) in the School of Biosciences and Chemistry were invited to take part in the study via online questionnaires. Staff were invited to complete the questionnaire via email and were signposted to the questionnaire in staff meetings. Students were invited to complete the questionnaire via email and in teaching sessions.

### Data analysis

Online questionnaire data were transferred to Excel, and quantitative bivariate analysis was undertaken. Short open responses were coded using qualitative content analysis, and frequency within responses was determined [22]. Employability skills were defined as communication, teamwork, leadership and related skills. Students were asked, ‘Thinking about subject knowledge, please describe the value of field trips within your degree?’, ‘Thinking about skills, please describe the value of field trips within your degree?’ and ‘Thinking about your course community, please describe the value of field trips within your degree?’ After initial analysis of the students’ responses, interplay between the three questions was observed, and thus responses across the three questions were coded together. Staff were asked, ‘What do you feel is the value of field trips in terms of subject knowledge, skills and sense of belonging?’ and initial codes were determined. Codes generated in the staff and student responses were considered together to form themes.

## Results

A questionnaire-based approach was taken to determine similarities and differences between staff and students’ perceptions of the impact of field trips. All academic staff and BSc undergraduate students were invited to take part in the study, with a 29% and 15% uptake rate, respectively.

**Figure 1:**
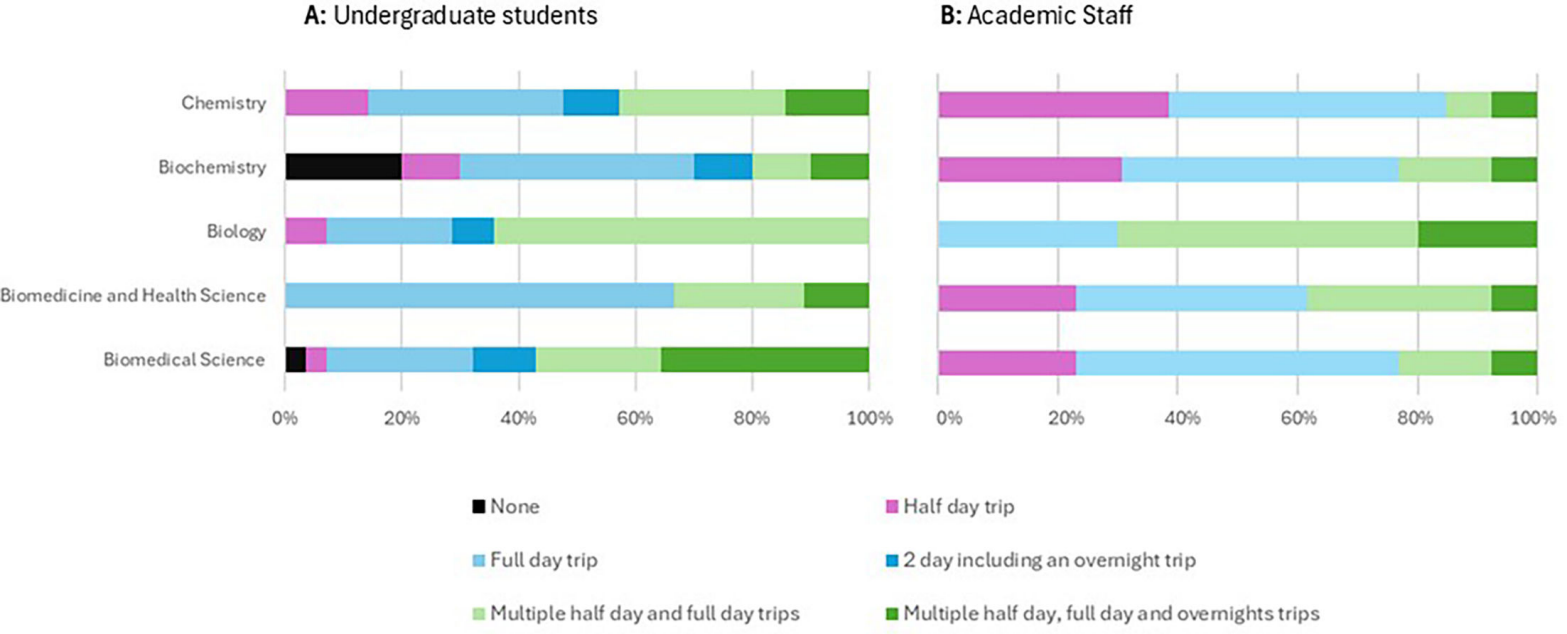
If you were to redesign the first year of your course, how many trips would you include?

To determine students’ desire for field trips in their undergraduate degrees, students were asked, ‘If you were to redesign the first year of your course, how many trips would you include?’ alongside their course of study. Staff were asked the related question, ‘If you were to redesign the first year of each of our current courses, how many trips would you include?’ (Figure 1).

Questionnaire data from undergraduate students and academic staff questionnaire shown as percentages. Chemistry (*n* = 21), Biochemistry, (*n* = 10), Biology (*n* = 14), Biomedicine and Health Science (*n* = 9), Biomedical Science (*n* = 28), Academic staff (*n* = 13).

Students’ desire for first-year field trips varied between courses. Biology students desired the most field trips in their course, with over 60% of respondents stating they would like to have multiple half and full-day trips in their first year of study. Biochemistry students desired the fewest trips, with 20% of students not wishing to attend any field trips in their first year.

Academic staff felt that all courses should have some field trip provision in their first year. Staff were relatively consistent in the number of trips they desired for each course, except for Biology, where a higher number of trips was desired.

### Knowledge, skills and a sense of belonging

To determine the perceived impact of field trips on students’ knowledge, skills and a sense of belonging, questionnaire open-text responses were coded by qualitative content analysis. Codes generated in the staff and student responses were considered together to form themes (Table 1).

**Table 1:** Perceived impact of field trips on students’ knowledge, skills and a sense of belonging

Theme	Student frequency (*n* = 65)	Staff frequency (*n* = 12)	Example text
Strengthen peer-to-peer relationships	34 (52%)	2 (17%)	‘A way for everyone to group together and get to know each other better’ [Student]
Insight into careers	28 (43%)	2 (17%)	‘We can see and understand how to apply our knowledge in workplace’ [Student]
Link theory to practice	28 (43%)	6 (50%)	‘Bridges the gap between theory knowledge and practical knowledge’ [Student]
Strengthen practical skills	27 (42%)	7 (58%)	‘Gives us more physical and practical skills’ [Student]
Deepens knowledge	26 (40%)	8 (67%)	‘Develop knowledge’ [Student]
Employability skills	24 (37%)	2 (17%)	‘Organisation skills increase and teamwork and communication and independence’ [Student]
Increases course belonging	9 (14%)	7 (58%)	‘I think it will instil a greater sense of community’ [Student]
Develops sense of being part of the wider scientific community	3 (5%)	1 (8%)	‘Allowing students to feel more inclined to want to become part of the community long term’ [Student]

Percentages do not equal 100% as open responses encompass more than one theme.

Over half of students (52%) felt that trips strengthen peer-to-peer relationships and 58% of academic staff saw trips as increasing course belonging, showing the value of field trips outside the taught curriculum. Both students and staff perceived trips to strengthen practical and employability skills as well as deepening knowledge and applying theory to practice.

### Accessibility

To determine the accessibility of trips, questionnaire open text responses were coded by qualitative content analysis. Students were asked, ‘Do/would you experience barriers to fully engaging with field trips? If so, please describe below’ and staff asked the related question ‘What are the barriers for students to fully engage with field trips?’ (Table 2).

**Table 2:** Student barriers to fully engaging with field trips

Theme	Student frequency (*n* = 55)	Staff frequency (*n* = 12)	Example text
No accessibility needs	30 (55%)	n/a	‘No I wouldn’t’ [Student]
Financial	14 (25%)	7 (58%)	‘Financial issues would be the main thing’ [Student]
Health concerns	6 (11%)	5 (42%)	‘Yes - mental health’ [Student]
Work commitments	3 (5%)	4 (33%)	‘Part-time work’ [Student]
Travel	2 (4%)	2 (17%)	‘Maybe travel in getting to places’ [Student]
Caring responsibility	0	3 (25%)	‘Caring responsibilities’ [Staff]

Percentages do not equal 100% as open responses may encompass more than one theme.

Both students and staff reported financial barriers as the most prevalent barrier to students fully accessing field trips, followed by health concerns and work commitments.

To better understand how to overcome these barriers, students and staff were asked, ‘How can we make field trips more accessible for you?’ and ‘What can we do to reduce students’ barriers to fully engage with field trips?’, respectively (Table 3).

**Table 3:** How to make field trips accessible

Theme	Student frequency (*n* = 51)	Staff frequency (*n* = 12)	Example text
Free/cheap	16 (32%)	2 (17%)	‘Make them free’ [Student]
Placement in the week	6 (12%)	3 (25%)	‘Put them during the working week when I’m definitely at university’ [Student]
Early communication	6 (12%)	3 (25%)	‘Make sure we know about them early enough’ [Student]
Provide travel	5 (10%)	2 (17%)	‘Provide a source of transport’ [Student]
Health considerations	1 (2%)	3 (25%)	‘Provide safe spaces in case the trip becomes too much’ [Student]
Cross-year peer support	0	2 (17%)	‘Talking to peers who have been on trips can encourage more participation’ [Staff]

Percentages do not equal 100% as open responses may encompass more than one theme.

Students’ most frequent measure to make field trips more accessible was based around cost, with 32% stating that they should be free or cheap, mirroring the prevalence of the cost barrier (Table 2). Students and staff both reported that trips should be in normal teaching times and be clearly communicated with students in a timely manner, mirroring the barrier themes of work and caring responsibilities (Table 2).

## Discussion

Field trips are valued by both staff and students for enhancing employability skills and subject knowledge and for fostering a sense of belonging and community. The learning benefits described are a) linking theory to practice, b) strengthening practical skills and c) deepening knowledge (Table 1). These are mentioned by both staff and students, though at higher rates by staff. These themes echo the learning benefits described in pedagogical literature, as field trips are known to benefit students’ ability to put theory into practice, learn in a real-life setting, make scientific connections and achieve high grades on associated coursework [1,2,12,13].

The most frequent theme in students’ responses to the impact of field trips was strengthening peer-to-peer relationships (52 %, *n* = 65, Table 1). Similarly, staff described their impact on increasing course belonging (58 %, *n* = 7, Table 1). Previous work often highlights the benefits of field trips for student socialisation; this was found in studies with explicit groupwork activities or simply with informal opportunities for social interactions [1,2,12,13]. Finally, students and staff highlighted transferable employability skills as well as the opportunity to gain insight into careers (Table 1). Not only can field trips benefit students’ transferable skills such as leadership, communication and professionalism [1,13], but a well-designed field trip can also provide knowledge on potential careers [13].

The majority of students on all courses desired some first-year field trip provision (Figure 1). While staff desired some first-year field trip provision across all courses, they desired a higher number of trips for Biologists (Figure 1). This is consistent with guidance from accrediting bodies and subject expectations for Biology [1,8]. It additionally shows that the potential benefits of field trips for students’ learning, transferable skills and socialisation were recognised more widely than simply in the environmental-based courses.

Rather than subject area, the main barriers that staff and students articulated were financial (Table 2). Field trips can be costly, especially those requiring extended travel and/or overnight accommodation [1,3,5]. How field trips are funded is key to their accessibility, with both students and staff stating that the cost is the main barrier to accessible field trips. UK universities are facing increasing financial challenges, adding additional complexities to how field trips are funded [23]. Students’ part-time work is also seen as a barrier to attending field trips [2]. In this study, both staff and students recommended that field trips be undertaken within university teaching time (Tables 2 and 3). This shows the complexities of student life, with some students’ current employment affecting their ability to engage with field trips, even though those trips would ultimately increase their employability skills and career awareness.

One potential solution lies in the use of virtual field trips, which have minimal or no cost, can be integrated either within class hours or flexibly within a student’s schedule, and allow students to explore inaccessible places [24]. However, students miss the sensory experience of being present at the site, and they may have decreased knowledge and enjoyment compared with traditional field trips [25].

Lei [1] proposes the use of campus field trips for biological teaching, where trips are accomplished either on campus or within a short walking distance [1]. While our study defined field trips as occurring off university premises, a field trip within easy walking distance of the university meets both our and Lei’s definition. Campus field trips have the benefit of fitting within the normal teaching time slots during the week to accommodate students’ jobs and having no costs for students or the institution [1,3], key inclusivity criteria identified by our students and staff (Table 3). Studies find that students report similar benefits from these forms of field trips as reported for other in-person trips; these trips were also associated with higher grades for participating students [4]. While Lei’s (2010) study focused on biology teaching [1], if relevant activities were designed for non-environmental courses, similar impacts might be expected; further study is required to test this hypothesis.

Even within a near-campus field trip, inclusion needs, such as for students with disabilities, must be considered when scouting out the location and designing the lesson plans [3]. Furthermore, regardless of the style of field trip chosen, early communication with students is key to ensuring accessibility (Table 3).

The results of this study demonstrate student and staff enthusiasm for field trips within a range of bioscience and chemistry degrees, alongside the practical realities of ensuring the developing field-trip offer is cost-effective, impactful and accessible.

## Data Availability

Data are available at 10.6084/m9.figshare.30093643

## Open Access

This article has been published open access under our Subscribe to Open programme, made possible through the support of our subscribing institutions, learn more here: https://portlandpress.com/pages/open_access_options_and_prices#conditional
